# Comparison of overfed Xupu and Landes geese in performance, fatty acid composition, enzymes and gene expression related to lipid metabolism

**DOI:** 10.5713/ajas.19.0842

**Published:** 2020-01-13

**Authors:** Xu Liu, Peng Li, Changqing He, Xiangyong Qu, Songchang Guo

**Affiliations:** 1 College of Animal Science and Technology, Hunan Agricultural University, Changsha, Hunan 410128, China; 2 Hunan Engineering Research Center of Poultry Production Safety, Hunan 410128, China; 3 Hunan Co-Innovation Center of Animal Production Safety, Changsha, Hunan 410128, China

**Keywords:** Xupu Geese, Landes Geese, Liver, Serum Parameters, Fatty Acid Composition, Genes Expression

## Abstract

**Objective:**

The aim of this study was to compare overfeeding performance, fatty acid composition, blood chemistry, enzymes and genes expression overfed Xupu and Landes geese.

**Methods:**

Sixty male Xupu geese (80 d) and Landes geese (80 d) were selected. After a period of one-week of pre-overfeeding, Xupu and Landes geese were overfed three meals of 550 and 350 g/d, respectively, of a high-carbohydrate diet in the first week of the overfeeding period. The next week, geese were given four meals of 1,200 and 850 g/d, respectively, over 8 to 14 d. Finally, geese were given five meals of 1,600 and 1,350 g/d, respectively, for the last two weeks.

**Results:**

After overfeeding for 28 d: Compared with Landes geese, Xupu geese liver weight and liver-to-body weight ratio decreased (p<0.05), while final weight, slaughter weight, total weight gain, abdominal fat weight, and feed-to-liver weight ratio increased (p<0.05). The levels of elaidic acid (C18:1t9), oleic acid (C18:1n-9), eicosenoic acid, and arachidonic acid in the liver of Xupu geese significantly increased (p<0.05), and the levels of myristic acid and stearic acid significantly decreased (p<0.05), while methyleicosanoate acid significantly increased (p<0.05). Xupu geese had higher plasma concentrations of triglyceride and very low density lipoprotein cholesterol (p<0.05), and decreased activities of alanine aminotransferase, aspartate aminotransferase, and lipase (LPS) (p<0.05). Landes geese had higher LPS activity (p<0.05), but lower cholinesterase activity (p<0.05) when compared with Xupu geese. The mRNA expression levels of fatty acid dehydrogenase (*FADS*) gene, elongase of long-chain fatty acid 1 (*ELOVL1*) gene, *ELOVL5*, and acyl-Co A: cholesterol acyltransferase 2 (*ACAT2*) gene were significantly upregulated (p<0.05) in Landes goose when compared with Xupu geese.

**Conclusion:**

This study demonstrates that the liver production performance of Landes geese was better than that of Xupu geese to some extent, which may be closely related to LPS activity, as well as the expression of *FADS*, *ELOVL1*, *ELOVL5*, and *ACAT2*.

## INTRODUCTION

With the occurrence of African swine fever in China, the consumption of safer poultry meat has increased dramatically [1,2]. When compared with other poultry meat, goose meat has beneficial characteristics including high protein and low fat content [3]. Moreover, goose liver has a high capacity of fat accumulation and is used for the production of “foie gras” in poultry production [4,5]. Unlike mammalian fatty liver, main components of foie gras are unsaturated fatty acids (UFA), which have been shown to help protect against cardiovascular and cerebrovascular disease in humans [6,7].

The Landes goose (*Anser anser*) originated in the southwestern Landes province of France. Landes geese have a beneficial liver capacity in that it can increase 5- to 10-fold in size over the course of a short-term overfeeding procedure. As a result, the Landes geese have become the world’s most famous breed for producing fatty liver products [8]. The Xupu goose (*Anser cygnoides domesticus*) is an indigenous breed of western Hunan Province in China and has been included in the list of National Livestock and Poultry Genetic Resources Protection in China [9,10]. When compared with other domestic goose breeds, the Xupu goose has the best capacity for fat accumulation in the liver. To this end, Fournier et al [11] showed that the difference of liver size between the two breeds of geese may be closely related to heredity. This difference may also be attributable to a wide range of other factors, including sex, nutrition, housing density, and housing environment.

At present, many studies have been conducted on Landes geese relative to Xupu geese; as a result, comparative studies between the two breeds remain scarce. Here, we sought to investigate overfeeding performance, plasma biochemistry indices, fatty acid composition, enzymes and gene expression related to lipid metabolism between Xupu and Landes geese. These data not only form the basis for the study regarding the mechanisms of liver fat deposition, but also provided a theoretical reference for the breeding of fatty liver geese.

## MATERIALS AND METHODS

### Experimental design, diets, and animal management

All experiment were approved by the Institutional Animal Care and Use Committee of the Hunan Agricultural University (Hunan, China). All methods and procedures were performed in accordance with the approved guidelines and provided by the regional Animal Ethics Committee.

Sixty male Xupu geese (4,947.00±377.54 g) and sixty male Landes geese (4,751.00±244.73 g) were selected for this study. All geese were maintained under the same feeding and management conditions. Xupu geese were provided by the Hunan Hongyu Xupu Goose Industry Development Co., Ltd. (Huaihua City, Hunan Province, P. R. China) and Landes geese were provided by the Hunan Fugoose Industry Development Co., Ltd. (Chenzhou City, Hunan Province, P. R. China). At 80 d of age, a period of one-week of pre-overfeeding began. During this time, food intake was progressively increased to enlarge the volume of the digestive tract and to initiate metabolic adaptation to overfeeding. At the end of the pre-overfeeding period, all geese were force-fed with a carbohydrate diet consisted of 98% boiled maize, 1.0% plant oil, 0.5% salt, and 0.5% multivitamin (multivitamin provided per kilogram of diet: vitamin A, 80,000,000 IU; vitamin D, 6,000,000 IU; vitamin E, 40,000 IU; vitamin B_1_, 12,000 mg; vitamin B_2_, 50,000 mg; vitamin B_6_, 500 mg; vitamin B_12_, 2,000 mg; vitamin K_3_, 3,000 mg; vitamin C, 16,000 mg; pantothenic acid, 3,000 mg; folic acid, 2,000 mg; nicotinic acid, 4,000 mg; biotin, 2,000 mg; methionine, 10,000 mg; lysine, 8,000 mg; tryptophan, 800 mg; arginine, 1,800 mg; serine, 8,000 mg; alanine, 18,000 mg). In total, this intake amounted to 3,370 kcal/kg, with a compasition of 90 g of protein/kg and 4.5 g of fat/kg. Xupu geese, having a greater capacity for overfeeding ingestion, were fed by the operator to the maximum of their ingestion potential. The feed intake of Xupu and Landes geese were different because of their different body weights. Xupu and Landes geese were overfed three meals of 550 and 350 g/d, respectively, of a high-carbohydrate diet in the first week of the overfeeding period. The next week, geese were given four meals of 1,200 and 850 g/d, respectively, over 8–14 d. Finally, geese were given five meals of 1,600 and 1,350 g/d, respectively, for the last two weeks.

This overfeeding experiment was conducted from July to August in 2018. The overfeeding room had a constant temperature (28°C to 34°C) and humidity (65% to 70%) range. All geese were divided into two groups according to the varieties and reared on the ground (4 m×6 m). Each goose was labeled with a ring on its right foot. An overfeeding machine was used in our study, and operated by the same person each time overfeeding occurred. During the overfeeding period, all goose had *ad libitum* access to water.

### Sample collection

Initial weight of each geese was recorded before the first meal of overfeeding. After 28 d of overfeeding, all geese were food deprived overnight for 12 h. During this time, geese had *ad libitum* access to water. On the following morning, geese were weighted and blood samples were taken by puncture of the occipital venous sinus. Blood sampling were maintained at room temperature for 1 h, and plasma was obtained by centrifugation at 3,000×g for 20 min at 4°C. After blood sampling, the goose were killed by exsanguination and each individual liver was quickly removed and weighed. Liver sample were immediately taken from the ventromedial portion of the main lobe (right lobe) of eight Xupu geese and eight Landes geese with similar body weight gain. Liver samples were immediately frozen in liquid nitrogen and stored at −80°C until later analysis of enzyme activities and mRNA levels. Initial weight, final weight, slaughter weight, liver weight were recorded daily by each goose to calculate the total weight gain, body weight gain rate, liver-to-body weight ratio, and feed-to-liver weight ratio. The indicator is calculated as follows:

$$\begin{array}{l} \text{Total weight gain}={\text{M}}_{\text{f}}-{\text{M}}_{\text{i}}\\ \text{Body weight gain rate}=({\text{M}}_{\text{f}}-{\text{M}}_{\text{i}})/{\text{M}}_{\text{i}}\times 100\%\\ \text{Liver-to-body weight ratio}={\text{M}}_{\text{l}}/{\text{M}}_{\text{s}}\times 100\%\\ \text{Feed-to-liver weight ratio}={\text{M}}_{\text{F}}/{\text{M}}_{\text{l}} \end{array}$$

M_f_, final weight; M_i_, initial weight; M_s_, slaughter weight; M_l_, liver weight; M_F_, total feed consumption of each goose during overfeeding.

### Fatty acid composition

The fatty acid composition of the liver was determined according to a previously described method [12]. Briefly, total lipids were extracted from the liver tissue using petroleum ether/anhydrous diethyl ether (1:1, v/v). Methyl esters of the lipids were prepared using saponification with a solution of KOH: methanol (4 mol:1 L). The organic layer was aspirated for fatty acid analysis using an Agilent 7890N gas chromatography equipped with a flame ionization detector (Agilent Technologies, Santa Clara, CA, USA) and a CP-Sil 88 fused silica open tube capillary column (100 m×0.25 nm; Agilent Technologies, USA). The gas chromatograph temperature program was as follows: Initial temperature of 140°C for 5 min, temperature increase of 3°C/min to 220°C, 1 min temperature hold at 220°C, and then holding temperature at 220°C for additional 40 min. The injector and detector temperatures were maintained at 240°C and 260°C, respectively. Hydrogen was used as the carrier gas at a flow rate of 40 mL/min. Individual fatty acid peaks were identified by comparing their retention times with those of the standards (Cat#: 18919-1AMP; Sigma Chemicals, St. Louis, MO, USA). The results were expressed as grams per 100 g of total identified fatty acids.

### Plasma biochemistry

Concentrations of triglyceride (TG), cholesterol (TC), low density lipoprotein cholesterol (LDL-C), very low density lipoprotein cholesterol (VLDL-C), and high density lipoprotein cholesterol (HDL-C) in plasma were measured using a Mindray automatic analyzer (BS-300; Shenzhen Mindray Bio-Medical Electronics Co., Ltd, Shenzhen, Guangdong, China) using a commercially available kits (Shenzhen Mindray Bio-Medical Electronics Co., Ltd., China) according to the manufacturer’s instructions. Aspartate aminotransferase (AST, C010-1) and alanine aminotransferase (ALT, C009-2) were determined using corresponding, commercially, available diagnostic kits (Nanjing Jiancheng Bioengineering Institute, Nanjing, China) using a microplate reader (Multiskan GO; Thermo Fisher Scientific, Waltham, CT, USA) according to the instructions of the manufacturer.

### Lipid metabolism enzymes activities of liver and plasma

Approximately 0.5 g of liver sample was used to prepare the tissue homogenate. Tissues were diluted in 1:9 (w/v) using ice-cold 154 mmol/L sodium chloride solution, and homogenized using an Ultra-Turrax homogenizer (T10BS25, IKA, Baden-Wurttemberg, Germany). Resulting homogenates were then centrifuged at 3,500×g at 4°C for 10 min.

The supernatant and plasma were used to determine the activities of cholinesterase (CHE), lipase (LPS), lipoporteinlipase (LPL), hepaticlipase (HL), and content of nonesterified free fatty acids (NEFA). All activities and content were determined according to corresponding, commercially available diagnostic kits (Nanjing Jiancheng Bioengineering Institute, China) according to the manufacturer’s instructions using a microplate reader (Multiskan GO; Thermo Fisher Scientific, USA). Protein concentration in the supernatant of the liver homogenate was measured by using a protein assay kit (A045-2; Nanjing Jiancheng Institute of Bioengineering, China).

### Total RNA extraction, reverse transcription, and quantitative real-time polymerase chain reaction

Total RNA was isolated from liver tissues using a TaKaRa MinBEST Universal RNA Extraction Kit (Takara, Osaka, Japan) according to the manufacturer’s protocol. The concentration and integrity of RNA were determined using a Nano-Drop 2,000 Spectrophotometer (Thermo Scientific, Hudson, NH, USA) and 1% agarose gel electrophoresis, respectively. Only RNA specimens with an A260/A280 ratio of 1.8 to 2.0 and an A260/A230 ratio ≥2.0 were used for subsequent analyses. Total RNA from each sample was reverse transcribed into cDNA using the PrimeScript RT reagent Kit with gDNA Eraser kit (Takara, Japan), and cDNA was then diluted 1:10 with nuclease-free water before being used for quantitative real-time polymerase chain reaction (PCR). The primer pairs for the amplification of adipocyte fatty acid binding protein (*FABP4*), stearoyl-CoA desaturase (*SCD*), fatty acid dehydrogenase (*FADS*), elongase of long-chain fatty acid 1 (*ELOVL1*), acyl-Co A: cholesterol acyltransferase 2 (*ACAT2*), LPL, fatty acid synthase (*FASN*), glyceraldehyde-3-phosphate dehydrogenase (*GAPDH*), and beta-actin (*β-actin*) gene were designed from GeneBank sequences using Primer Premier 5.0 and obtained from Shanghai Sheng-Gong Biological Company (Shanghai, China), as shown in Table 1.

Quantitative real-time PCR (qPCR) was performed using SYBR Green Master Mix (Vazyme Biotech, Nanjing, China) in CFX96 Touch Real-Time PCR Detection System (Bio-Rad Laboratories, Hercules, CA, USA). The PCR systems consisted of 2 μL of diluted cDNA template (1:9), 12.5 μL of SYBR Premix Ex Taq II, 1 μL PCR Forward Primer (10 μmol/L), 1 μL PCR Reverse Primer (10 μmol/L) and 8.5 μL of sterilized distilled water. The PCR programs was as follows: 95°C for 30 s, followed by 35 cycles of denaturation at 95°C for 5 s and 60°C for 30 s. Dissociation curves of the products were generated by increasing the temperature of samples incrementally from 55°C to 95°C as the final step of the PCR. *GAPDH* and *β-actin* genes were used as the dual internal standard for normalizing transcript abundance of mRNA expression. The relative expression levels of target genes were calculated by the 2^−ΔΔCt^ method as described by Livak and Schmittgen [13].

### Statistical analyses

All data were analyzed using SPSS 21.0 (2015, IBM-SPSS Inc., Chicago, IL, USA). Variability of all the data is expressed as standard error of the mean (SEM). Differences between mean values were compared using independent samples t-test, and considered significant at p<0.05.

## RESULTS

### Overfeeding performance

The results of overfeeding performance of Xupu and Landes geese are presented in Table 2 and 3. When compared with the Landes geese, final weight, total weight gain, slaughter weight, abdominal fat weight and feed-to-liver weight ratio of Xupu geese significantly increased (p<0.05). In Xupu geese, the liver weight and liver-to-body weight ratio both decreased (p<0.05). There were no significant differences in the initial weight and body weight gain rate between Xupu and Landes geese (p>0.05).

### Fatty acid composition

The fatty acid composition in liver of Xupu and Landes geese are shown in Table 4. Analyses of liver fatty acids showed that the major fatty acids were palmitic acid (C16:0), stearic acid (C18:0), palmitoleic acid (C16:1), oleic acid (C18:1n-9), and linoleic acid (C18:2n-6c). Oleic acid (C18:1n-9) was the most abundant fatty acid, accounting for 58.25% and 54.82% of total fatty acid in livers of Xupu and Landes geese, respectively. When compared with the Landes geese, the levels of myristic acid (C14:0) and stearic acid (C18:0) decreased (p< 0.05) in the livers of Xupu geese, contrastingly, levels of methyleicosanoate acid (C20:0) increased (p<0.05). In the livers of Xupu geese, the levels of elaidic acid (C18:1t9), oleic acid (C18:1n-9), eicosenoic acid (C20:1), and arachidonic acid (C20:4n-6) significantly increased (p<0.05). However, no significant differences were found in palmitic acid (C16:0), palmitoleic acid (C16:1), or linoleic acid (C18:2n-6) between either Xupu or Landes geese (p>0.05).

### Blood chemistry

As is shown in Table 5, the results of blood chemistry analyses for Xupu and Landes geese revealed that there were no significant differences in either TC or LDL-C (p>0.05). However, plasma levels of TG and VLDL-C were significantly higher (p<0.05) in Xupu geese when compared with Landes geese. When compared with Landes geese, Xupu geese had a significant decrease (p<0.05) in HDL-C content, as well as ALT and AST activities.

### Lipid metabolism-related enzymes activities

The results of lipid metabolism-related enzymes activities in the plasma and liver of Xupu and Landes geese are presented in Table 6. When compared with the Landes geese, LPS activity in the plasma and liver of Xupu geese both significantly decreased (p<0.05). However, a significant enhancement (p<0.05) of CHE activity in the liver of Xupu geese was observed. There were no significant differences in either plasma or liver LPL or HL activities or in the NEFA content between Xupu and Landes geese (p>0.05).

### Gene expression

The lipid metabolism-related gene mRNA expression in livers of Xupu and Landes geese are shown in Table 7. The mRNA expression of *FADS*, *ELOVL1*, *ELOVL5*, and *ACAT2* (p<0.05) in liver of Xupu goose were significantly downregulated than in liver of Landes goose. There were no significant differences in the mRNA expression of *FABP4*, *SCD*, *LPL*, *FASN*, and retinol binding protein 7 in liver between Xupu and Landes geese (p<0.05).

## DISCUSSION

In our study, we found that the livers of Xupu geese were smaller and the feed-to-liver weight ratio of Xupu geese were higher than those of Landes geese. Since Xupu geese had a larger body shape, the index of liver-to-body weight ratio revealed an opposite result. The abdominal fat weight of Xupu geese was higher than that of Landes geese. Possible reasons for this result include that a large amount of fat was transferred from the liver to extrahepatic (*e.g.*, abdominal adipose tissue) in Xupu geese. The above results confirmed that Landes geese are the best breed globally to produce fatty liver products.

Overfeeding with a carbohydrate-rich diet results in high *de novo* lipogenesis. The present study was the first time to compare the fatty acid composition of the fatty liver in Landes and Xupu geese. There were both similarities and differences between the two breeds. The main products of fatty acid synthesis were 16:0, 18:0, and above all 18:1. This fatty acid in particular accounted for more than 90% of the hepatic TG fatty acid content, and the proportions of 18:1 was more than 50% in both breeds. These results were consistent with the fatty liver content found previously in both geese and duck [14,15]. Relative to Landes geese, Xupu geese had an increase in the proportions of 18:1, but a decrease in the proportions of 18:0 in their fatty liver. This finding was in agreement with those found by both Cazeils et al [16] and Hatsugai et al [17]. Simultaneously, the proportions of monounsaturated fatty acids and polyunsaturated fatty acids (PUFA) in the fatty liver of Xupu geese were significantly higher than those in Landes geese. Moreover, the proportion of saturated fatty acids in the fatty liver of Xupu geese was significantly lower than that of Landes geese. These results were consistent with the previous study, which used 21 days of feeding for both Xupu and the Landes geese [18]. Given these findings, it is clear that the performance of overfeeding production and fat deposition in Xupu geese were inferior to that of Landes geese. However, the proportions of UFA in fatty liver of Xupu geese was significantly higher than that of Landes geese, which may indicate that Xupu fatty liver is better for human health.

Fatty liver occurs in geese or duck when fat synthesis exceeds fat secretion. In response to overfeeding, *de novo* hepatic lipogenesis is dramatically increased, TG do not fully enter the secretion pathway, and a large proportion of TG remains stored in the liver [11,19]. During overfeeding, part of the newly synthesized TG in the liver are incorporated into hepatic lipoprotein, mainly VLDL which can be secreted into blood and used (or stored) in extrahepatic tissues. We found that the plasma contents of TG and VLDL of Xupu geese were higher than those of Landes geese. These results were in agreement with previous studies, which showed higher VLDL concentration in Poland geese when compared with Landes geese, even though the liver weight of the former was less than the latter [11]. Xu et al [20] found that the plasma concentrations of TG and VLDL were higher in Sichuan white geese than in Landes geese. In the present study, overfeeding with high-energy corn diet for 28 d induced elevations in the concentration of plasma HDL, which was in accordance with findings of previous studies in broiler chickens and geese [21,22]. It clear the transfer of fat from the liver to the extrahepatic tissue is one of the reasons why the liver weight of Xupu geese was smaller, but their abdominal fat heavier than that of Landes geese. These results suggested that the mechanism behind geese fatty liver formation is mainly attributable to an imbalance between the storage and secretion (as plasma lipoproteins) of newly synthesized endogenous lipids and exogenous lipids in the cytoplasm.

When overfeeding using a high-energy corn diet, plasma ALT and AST are mainly from the liver, resulting in higher ALT and AST activities. Our study found that the plasma activities of ALT and AST in Xupu geese were lower than those of Landes geese, indicating that Landes geese had a higher degree of liver damage due to long-term overfeeding. The results in this experiment were in agreement with those of Zhu et al [21], which showed that long-term overfeeding induced liver cell inflammation. Therefore, we supported the opinion that goose hepatic adaptation to overfeeding is of notable importance. Our study found that the activity of LPS in the plasma and liver of Landes geese increased relative to that of Xupu geese. Pancreatic lipase plays an important role in fat absorption. Kobayashi et al [23] showed that lipase activity was high by using the higher fat content of diets. Krogdahl [24] also reported that when given a high-fat diet, the lipase activity of birds was higher than that in birds fed a low-fat diet. Much past work has found that the activity of LPL was positively correlated with the weight of fatty liver in goose or duck, moreover, that LPL was useful in the selection of Landes geese breeders with a higher susceptibility to liver steatosis [6,25,26]. Despite this, we found no difference in the activity of LPL in either plasma or liver between Xupu and Landes geese, suggesting further study is needed.

Collectively, our previous study [18] and current data indicate the development of severe steatosis was closely related to the genes involved in lipid synthesis, packaging, secretion, transportation, deposition or metabolism, including *FADS*, *ELOVL1*, *ELOVL5*, and *ACAT2* [27,28]. It is well known that *FADS* gene plays a key role in the synthesis of long-chain polyunsaturated fatty acid (LC-PUFAs) and the metabolism of essential fatty acids [29–33]. ELOVL1 plays an important role in the elongation of super-long-chain saturated fatty acids and super-long-chain monosaturated fatty acids. ELOVL5 is mainly responsible for the elongation of 18-carbon fatty acids. ACAT2 catalyzes the conjugation of cholesterol and long-chain fatty acids to form cholesterol esters, and plays an important role in the absorption, storage, transport, and apolipoprotein metabolism of cholesterol. Given this, we observed that the expression of *FADS*, *ELOVL1*, *ELOVL5*, and *ACAT2* in the liver of Landes geese increased significantly relative to Xupu geese. Osman et al [34] showed that *FADS* expression levels were gradually increased after overfeeding, moreover, that the induction of *FADS* promoted the generation of LC-PUFAs in goose fatty liver. This increase appeared to be well coordinated with the size of fatty liver in Landes geese, which suggests that the *FADS*, *ELOVL1*, *ELOVL5*, and *ACAT2* genes are important to the development of goose fatty liver.

In conclusion, the results of the present study showed that the overfeeding performance of Xupu geese was inferior to that of Landes geese, which may be related to the activity of LPS, and the expression of *FADS*, *ELOVL1*, *ELOVL5*, and *ACAT2*. However, the the proportions of UFA in Xupu fatty liver were significantly higher than those of Landes geese. Taken together, there results provide new insights into the cultivation of high-quality fatty liver geese.

## Figures and Tables

**Table 1:** The primers used for gene cloning, quantitative real-time polymerase chain reaction

Gene name	Accession number	Forward sequence (5′ to 3′)	Reverse sequence (5′ to 3′)
*FABP4*	HQ833815.1	CAAGCCCAATGTAACTATCA	ATCAAACTCTTCACCCAACT
*SCD*	HQ197924.1	CTCGGTGGTGTTGCTGTGCT	GGTTCTCCCGTGGGTTGAT
*FADS*	XM013177620.1	ATCTTTCAGTCTTCAGCGA	ACAGAGAGTGTTTTTCCCA
*ELOVL1*	XM013177961.1	TTTGGACCAGGGGGAA	GGAGATGTGGACAGAGACG
*ELOVL5*	XM_013172275.1	ACACCTACCTTTGTCTTCTC	TCAGCTTCACCTCCACT
*ACAT2*	XM013186797.1	GGAACTGGGACGGTAAC	GAGCCAAAGGCATAAGA
*LPL*	XM013188252.1	TACCTGAAGACCCGTGC	ACTCTCATCTAAAGTGCCATAC
*FASN*	XM013197939.1	AATCCAGAAGGGCCAAC	TCCAGCAATGCGGTAAG
*RBP7*	XM_013194243.1	TCGCAACACGCAAGATA	TCAAGCCAGTGAGTCCA
*GAPDH*	XM027449739.1	GCCCAGAACATTATCCCA	CAGGTCAGGTCCACGACA
*β-actin*	M26111.1	CGAGCGGTTCAGGTGTCCA	CGTCGTATTCCTGCTTGCTG

*FABP4*, adipocyte fatty acid binding protein; *SCD*, stearoyl-CoA desaturase; *FADS*, fatty acid dehydrogenase; *ELOVL1*, elongase of long-chain fatty acid 1; *ELOVL5*, elongase of long-chain fatty acid 5; *ACAT2*, cholesterol acyltransferase 2; *LPL*, lipoprotein lipase; *FASN*, fatty acid synthase; *RBP7*, retinol binding protein 7; *GAPDH*, glyceraldehyde-3-phosphate dehydrogenase; *β-actin*, beta-actin.

**Table 2:** Comparison of performance parameters between overfed Xupu and Landes geese

Items	Xupu geese	Landes geese	SEM	p-value
Initial weight (g)	4,947.00	4,851.00	100.606	0.120
Final weight (g)	8,843.00^a^	8,031.00^b^	194.983	<0.001
Total weight gain (g)	3,896.00^a^	3,280.00^b^	93.726	0.001
Body weight gain rate (%)	78.74	74.14	3.017	0.139

Data are means of 8 geese per group.

SEM, standard error mean.

a,b Means within a row with different superscripts differ significantly (p<0.05).

**Table 3:** Comparison of slaughter traits between overfed Xupu and Landes geese

Items	Xupu geese	Landes geese	SEM	p-value
Slaughter weight (g)	8,154.75^a^	7,188.25^b^	208.733	<0.001
Liver weight (g)	609.08^b^	938.40^a^	58.886	<0.001
Abdominal fat weight (g)	557.25^a^	360.45^b^	32.814	<0.001
Liver-to-body weight ratio (%)	7.47^b^	13.07^a^	0.731	<0.001
Feed-to-liver weight ratio (kg·kg)	44.44^a^	22.82^b^	3.083	<0.001

Data are means of 8 geese per group.

SEM, standard error mean.

a,b Means within a row with different superscripts differ significantly (p<0.05).

**Table 4:** Comparison of fatty acid composition between overfed Xupu and Landes geese

Items	Xupu geese	Landes geese	SEM	p-value
Myristic acid (C14:0)	0.46^b^	0.60^a^	0.056	0.030
Palmitic acid (C16:0)	23.30	25.17	0.989	0.088
Stearic acid (C18:0)	12.54^b^	15.03^a^	0.457	<0.001
Methyleicosanoate acid (C20:0)	0.21^a^	0.14^b^	0.009	<0.001
SFA (%)	36.38^b^	40.94^a^	1.118	0.002
Palmitoleic acid (C16:1)	2.50	2.11	0.234	0.149
Elaidic acid (C18:1t9)	0.36^a^	0.31^b^	0.017	0.048
Oleic acid (C18:1n-9c)	58.25^a^	54.82^b^	1.135	0.015
Eicosenoic acid (C20:1)	0.24^a^	0.15^b^	0.009	<0.001
MUFA (%)	60.91^a^	57.38^b^	1.005	0.006
Linoleic acid (C18:2n-6c)	1.48	1.21	0.214	0.279
Arachidonic acid (C20:4n-6)	0.89^a^	0.47^b^	0.177	0.049
PUFA (%)	2.32^a^	1.68^b^	0.363	0.017

Data are means of 8 geese per group.

SEM, standard error mean; SFA, saturated fatty acids; MUFA, monounsaturated fatty acids; PUFA, polyunsaturated fatty acids.

a,b Means within a row with different superscripts differ significantly (p<0.05).

**Table 5:** Comparison of plasma biochemical metabolites between overfed Xupu and Landes geese

Items	Xupu geese	Landes geese	SEM	p-value
TG (mmol/L)	6.04^a^	4.33^b^	0.378	0.001
TC (mmol/L)	10.42	11.51	0.659	0.125
LDL-C (mmol/L)	3.13	3.47	0.280	0.245
VLDL-C (mmol/L)	0.92^a^	0.52^b^	0.151	0.026
HDL-C (mmol/L)	6.12^b^	7.24^a^	0.353	0.009
ALT (U/L)	50.97^b^	94.68^a^	5.181	<0.001
AST (U/L)	87.73^b^	234.98^a^	7.199	<0.001

Data are means of 8 geese per group.

SEM, standard error mean; TG, triglyceride; TC, cholesterol; LDL-C, low density lipoprotein cholesterol; VLDL-C, very low density lipoprotein cholesterol; HDL-C, high density lipoprotein cholesterol; ALT, alanine aminotransferase; AST, aspartate aminotransferase.

a,b Means within a row with different superscripts differ significantly (p<0.05).

**Table 6:** Comparison of enzyme activities in blood and liver between overfed Xupu and Landes geese

Items	Xupu geese	Landes geese	SEM	p-value
Plasma				
CHE (U/mL)	330.12	342.50	64.194	0.851
LPS (U/L)	16.40^b^	27.81^a^	3.854	0.034
LPL (U/mL)	5.58	5.84	0.447	0.567
HL (U/mL)	5.62	5.62	0.650	0.995
LPL+HL (U/mL)	10.67	11.45	0.873	0.385
NEFA (U/mL)	490.97	470.77	93.500	0.833
Liver				
CHE (U/mgprot)	26.77^a^	7.41^b^	7.038	0.022
LPS (U/gprot)	66.70^b^	139.77^a^	21.311	0.027
LPL (U/mgprot)	0.46	0.56	0.137	0.505
HL (U/mgprot)	0.45	0.45	0.265	0.990
LPL+HL (U/mgprot)	0.84	1.01	0.296	0.562
NEFA (U/gprot)	66.48	56.04	14.670	0.497

Data are means of 8 geese per group.

SEM, standard error mean; CHE, cholinesterase; LPS, lipase; LPL, lipoporteinlipase; HL, hepaticlipase; NEFA, nonesterified free fatty acids.

a,b Means within a row with different superscripts differ significantly (p<0.05).

**Table 7:** Comparison of lipid metabolism related gene expressions in liver between overfed Xupu and Landes geese

Items	Xupu geese	Landes geese	SEM	p-value
*FABP4*	1.00	0.91	0.326	0.838
*SCD*	1.00	1.15	0.156	0.777
*FADS*	1.00^b^	3.16^a^	0.335	0.044
*ELOVL1*	1.00^b^	3.16^a^	0.210	0.007
*ELOVL5*	1.00^b^	2.36^a^	0.320	0.047
*ACAT2*	1.00^b^	2.40^a^	0.237	0.027
*LPL*	1.00	1.37	0.354	0.107
*FASN*	1.00	1.47	0.458	0.256
*RBP7*	1.00	1.53	0.212	0.242

Data are means of 8 geese per group.

SEM, standard error mean; *FABP4*, adipocyte fatty acid binding protein; *SCD*, stearoyl-CoA desaturase; *FADS*, fatty acid dehydrogenase; *ELOVL1*, elongase of long-chain fatty acid 1; *ELOVL5*, elongase of long-chain fatty acid 5; *ACAT2*, cholesterol acyltransferase 2; *LPL*, lipoprotein lipase; *FASN*, fatty acid synthase; *RBP7*, retinol binding protein 7.

a,b Means within a row with different superscripts differ significantly (p<0.05).

## References

[1] Galindo I, Alonso C (2017). African swine fever virus: a review. Viruses.

[2] Costard S, Mur L, Lubroth J, Sanchez-Vizcaino JM, Pfeiffera DU (2013). Epidemiology of African swine fever virus. Virus Res.

[3] Su SY, Dodson MV, Li XB, Li QF, Wang HW, Xie Z (2009). The effects of dietary betaine supplementation on fatty liver performance, serum parameters, histological changes, methylation status and the mRNA expression level of Spot14α in Landes goose fatty liver. Comp Biochem Physiol Part A Mol Integr Physiol.

[4] Liu L, Zhao X, Wang Q (2016). Prosteatotic and protective components in a unique model of fatty liver: gut microbiota and suppressed complement system. Sci Rep.

[5] Geng TY, Yang B, Li FY (2016). Identification of protective components that prevent the exacerbation of goose fatty liver: characterization, expression and regulation of adiponectin receptors. Comp Biochem Physiol B Biochem Mol Biol.

[6] Lu LZ, Chen Y, Wang Z (2015). The goose genome sequence leads to insights into the evolution of waterfowl and susceptibility to fatty liver. Genome Biol.

[7] De Souza RJ, Mente A, Maroleanu A (2015). Intake of saturated and trans unsaturated fatty acids and risk of all cause mortality, cardiovascular disease, and type 2 diabetes: systematic review and meta-analysis of observational studies. BMJ.

[8] Mourot J, Guy G, Lagarrigue S, Peiniau P, Hermier D (2000). Role of hepatic lipogenesis in the susceptibility to fatty liver in the goose (*Anser anser*). Comp Biochem Physiol B Biochem Mol Biol.

[9] Lin Q, Cao R, Jiang GT (2016). The complete mitochondrial genome of the Xupu goose. Mitochondrial DNA A.

[10] Dai QZ, Lin Q, Jiang GT (2016). Phylogenetic studies of four *Anser cygnoides* (Anserini: Anserinae) in Hunan province of China based on complete mitochondrial DNA sequences. Mitochondrial DNA A DNA Mapp Seq Anal.

[11] Fournier E, Peresson R, Guy G, Hermier D (1997). Relationships between storage and secretion of hepatic lipids in two breeds of geese with different susceptibility to liver steatosis. Poult Sci.

[12] Li FN, Duan YH, Li YH (2015). Effects of dietary *n*-6:*n*-3 PUFA ratio on fatty acid composition, free amino acid profile and gene expression of transporters in finishing pigs. Br J Nutr.

[13] Livak KJ, Schmittgen TD (2001). Analysis of relative gene expression data using real-time quantitative PCR and the 2^−ΔΔCt^ method. Methods.

[14] Hermier D, Salichon MR, Guy G, Peresson R (1999). Differential channelling of liver lipids in relation to susceptibility to hepatic steatosis in the goose. Poult Sci.

[15] Molee W, Bouillieroudot M, Auvergne A, Babilé R (2005). Changes in lipid composition of hepatocyte plasma membrane induced by overfeeding in duck. Comp Biochem Physiol B, Biochem Mol Biol.

[16] Cazeils JL, Bouillier-Oudot M, Auvergne A, Candau M, Babile R (1999). Lipid composition of hepatocyte plasma membranes from geese overfed with corn. Lipids.

[17] Hatsugai K, Ohkohchi N, Fukumori T, Akamatsu Y, Satomi S (2000). Mechanism of primary graft non-function in a rat model for fatty liver transplantation. Transpl Int.

[18] Liu X, Liu YW, He CQ (2019). Comparison of nutritional components and serum biochemical indices in fatty liver between Xupu geese and Landes geese. J Anim Nutr (in chinese).

[19] Hermier D, Saadoun A, Salichon MR, Sellier N, Rousselot-Paillet D, Chapman MJ (1991). Plasma lipoproteins and liver lipids in two breeds of geese with different susceptibility to hepatic steatosis: changes induced by development and force-feeding. Lipids.

[20] Xu HY, Wang Y, Han CC (2010). Estimation of lipoprotein-lipase activity (LPL) and other biochemical changes in two breeds of overfeeding geese. Asian-Australas J Anim Sci.

[21] Zhu LH, Meng H, Duan XJ, Xu GQ, Zhang J, Gong DQ (2011). Gene expression profile in the liver tissue of geese after overfeeding. Poult Sci.

[22] Peebles ED, Cheaney JD, Brake JD, Boyle CR, Latour MA, McDaniel CD (1997). Effects of added lard fed to broiler chickens during the starter phase. 2. Serum lipids. Poult Sci.

[23] Kobayashi S, Terashima Y, Itoh H (2002). Effects of dietary chitosan on fat deposition and lipase activity in digesta in broiler chickens. Br Poult Sci.

[24] Krogdahl A (1985). Digestion and absorption of lipids in poultry. J Nutr.

[25] Davail S, Guy G, Andre J, Hermier D, Hoo-Paris R (2000). Metabolism in two breeds of geese with moderate or large overfeeding induced liver-steatosis. Comp Biochem Physiol Part A Mol Integr Physiol.

[26] André JM, Guy G, Gontierlatonnelle K (2007). Influence of lipoprotein-lipase activity on plasma triacylglycerol concentration and lipid storage in three genotypes of ducks. Comp Biochem Physiol Part A Mol Integr Physiol.

[27] Sassa T, Ohno Y, Suzuki S (2013). Impaired epidermal permeability barrier in mice lacking Elovl1, the gene responsible for very-long-chain fatty acid production. Mol Cell Biol.

[28] Shikama A, Shinozaki H, Takeuchi Y (2015). Identification of human *ELOVL5* enhancer regions controlled by SREBP. Biochem Biophys Res Commun.

[29] Burdge GC, Calder PC (2005). Conversion of alpha-linolenic acid to longer-chain polyunsaturated fatty acids in human adults. Reprod Nutr Dev.

[30] Koletzko B (2001). Fatty acids and early human growth. Am J Clin Nutr.

[31] Molto-Puigmarti C, Plat J, Mensink RP (2010). *FADS1 FADS2* gene variants modify the association between fish intake and the docosahexaenoic acid proportions in human milk. Am J Clin Nutr.

[32] Arterburn LM, Hall EB, Oken H (2006). Distribution, interconversion, and dose response of n-3 fatty acids in humans. Am J Clin Nutr.

[33] Park WJ, Kothapalli KS, Reardon HT, Lawrence P, Qian SB, Thomas Brenna J (2012). A novel *FADS1* isoform potentiates FADS2-mediated production of eicosanoid precursor fatty acids. J Lipid Res.

[34] Osman RH, Liu L, Xia LL (2016). *Fads1* and *2* are promoted to meet instant need for long-chain polyunsaturated fatty acids in goose fatty liver. Mol Cell Biochem.

